# Insulin Regulates GABA_A_ Receptor-Mediated Tonic Currents in the Prefrontal Cortex

**DOI:** 10.3389/fnins.2018.00345

**Published:** 2018-05-31

**Authors:** Saraí Trujeque-Ramos, Diego Castillo-Rolón, Elvira Galarraga, Dagoberto Tapia, Gabina Arenas-López, Stefan Mihailescu, Salvador Hernández-López

**Affiliations:** ^1^Departamento de Fisiología, Facultad de Medicina, Universidad Nacional Autónoma de Mexico, Mexico City, Mexico; ^2^División de Neurociencias, Instituto de Fisiología Celular, Universidad Nacional Autónoma de Mexico, Mexico City, Mexico

**Keywords:** extrasynaptic GABA_A_ receptors, ambient GABA, receptor trafficking, brain slices, neuronal excitability

## Abstract

Recent studies, have shown that insulin increases extrasynaptic GABA_A_ receptor-mediated currents in the hippocampus, causing alterations of neuronal excitability. The prefrontal cortex (PFC) is another brain area which is involved in cognition functions and expresses insulin receptors. Here, we used electrophysiological, molecular, and immunocytochemical techniques to examine the effect of insulin on the extrasynaptic GABA_A_ receptor-mediated tonic currents in brain slices. We found that insulin (20–500 nM) increases GABA_A_-mediated tonic currents. Our results suggest that insulin promotes the trafficking of extrasynaptic GABA_A_ receptors from the cytoplasm to the cell membrane. Western blot analysis and immunocytochemistry showed that PFC extrasynaptic GABA_A_ receptors contain α-5 and δ subunits. Insulin effect on tonic currents decreased the firing rate and neuronal excitability in layer 5–6 PFC cells. These effects of insulin were dependent on the activation of the PI3K enzyme, a key mediator of the insulin response within the brain. Taken together, these results suggest that insulin modulation of the GABA_A_-mediated tonic currents can modify the activity of neural circuits within the PFC. These actions could help to explain the alterations of cognitive processes associated with changes in insulin signaling.

## Introduction

GABA_A_ receptors are the main inhibitory ligand-gated ion channels in the adult mammalian central nervous system (CNS). They are pentamers constituted by different subunits (α1-6, β1-3, γ1-3, ρ1-3, π, ε, δ, and θ) (McKernan and Whiting, 1996) forming an anion selective pore. The subunit composition of these receptors determines their kinetics and pharmacological properties (Macdonald and Olsen, 1994; Vicini and Ortinski, 2004). GABA_A_ receptors are either located at the synapses (synaptic) or outside the synapses (extrasynaptic) where they mediate phasic and inhibitory tonic currents respectively (Birnir and Korpi, 2007). Synaptic GABA_A_ receptors contain a γ2 subunit in association with α1, α2, or α3 subunits (α1β2/3γ2, α2β2/3γ2, and α3β2/3γ2), have lower affinity for GABA than those containing the same α subunits combined with the δ subunit (Stell and Mody, 2002; Farrant and Nusser, 2005). Synaptic receptors desensitize or inactivate rapidly (Bianchi et al., 2002; Farrant and Nusser, 2005) and mediate transient inhibitory postsynaptic currents (IPSCs) that regulate spike timing (Pouille and Scanziani, 2001). On the other hand, extrasynaptic GABA_A_ receptors contain α4, α5, α6, or δ subunits (α6βxδ, α4βxδ, and α5βxγ2) (Farrant and Nusser, 2005), exhibit high affinity for GABA and little or no desensitization (Farrant and Nusser, 2005). Extrasynaptic GABA_A_ receptors generate inhibitory tonic currents and are activated by the ambient GABA which reaches these receptors by diffusing from the synaptic sites of neighboring neurons (Rossi et al., 2003). The persistent inhibition mediated by extrasynaptic GABA_A_ receptors contributes to regulate the excitability of neural networks (Semyanov et al., 2003). Several studies have demonstrated that insulin has neuromodulator functions in the CNS. One of the most interesting effects of insulin is the modulation of GABA_A_ currents. At this respect, it has been reported that insulin promotes membrane translocation and clustering of synaptic GABA_A_ receptors and an increase of GABA_A_ mediated currents in hippocampal CA1 pyramidal neurons (Wan et al., 1997). In a more recent study, it was found that insulin also increases GABA_A_-mediated tonic currents in hippocampal CA1 pyramidal neurons (Jin et al., 2011). Tonic inhibition of hippocampal neurons is thought to regulate cognitive functions (Crestani et al., 2002; Caraiscos et al., 2004; Pavlov et al., 2009; Martin et al., 2010). However, insulin may modulate the neuronal activity in many other brain structures by the same mechanism. Autoradiographic studies have suggested that there is a high density of insulin receptors in several brain regions including the olfactory bulb and closely related limbic regions, basal ganglia, cerebellum and neocortex, among others (Hill et al., 1986; Werther et al., 1987). Here, we investigated the effect of insulin on pyramidal neurons of prefrontal cortex (PFC) a structure involved in cognitive, sensorial and emotional processes. We found that insulin increases tonic currents in pyramidal PFC neurons. Our data indicate that insulin promotes the trafficking of extrasynaptic GABA_A_ receptors to the cell membrane. We provide evidence that tonic currents are mediated by two separate subtypes of extrasynaptic GABA_A_ receptors containing either the α5 or the δ subunit.

## Materials and methods

### Slice preparation

All experiments were carried out in accordance with the National Institutes of Health Guide for the Care and Use of Laboratory Animals and were approved by the Institutional Animal Care Committee of the National Autonomous University of Mexico. Experiments were performed in young male (postnatal day 25–30) Wistar rats. Animals were deeply anesthetized with isoflurane and then decapitated. Their brains were quickly removed and placed into ice-cold (4°C) artificial cerebrospinal fluid (ACSF) consisting of (in mM): 125 NaCl, 3 KCl, 25 NaHCO_3_, 1.25 NaH_2_PO_4_, 1 MgCl_2_, 1.2 CaCl_2_, and 25 glucose, 300 mOsm, pH 7.3 by bubbling with 95% O_2_-5% CO_2_. Coronal brain slices (350 μm thick) containing the prefrontal cortex were obtained with a Vibratome (Pelco 102, Ted Pella. INC) and stored in oxygenated ACSF at room temperature for at least 1 h before recordings.

### Whole cell recordings

Individual slices were transferred into a custom-made Plexiglas recording chamber and perfused with ACSF at a rate of 4–5 ml/min, maintained at 33°C by an in-line solution heater (TC-324B; Warner Instruments). Layer 5–6 pyramidal neurons were visualized with an infrared videomicroscopy system (BX51WI; Olympus Instruments) fitted with an 80X water-immersion objective. The image from the microscope was obtained with a CCD camera and displayed on a monitor. Whole cell current and voltage-clamp recordings were performed with a Multiclamp 700B amplifier (Molecular Devices) and monitored with a PC running Clampex 10 software (Molecular Devices). Only one cell was recorded per brain slice. Micropipettes (5–7 MΩ) used for recordings, were made from borosilicate glass tubes (WPI, Sarasota, FL) with a Flaming-Brown puller (Sutter Instrument, Novato, CA). Experimental data were digitized and stored in a PC by using a Digidata 1440A analog-to-digital converter (Molecular Devices) at a sampling rate of 5 kHz. The internal solution for voltage-clamp recordings consisted of (in mM): 140 CsCl, 0.1 CaCl_2_, 1 EGTA, 0.5 KCl, 1 MgCl_2_, 2 ATP-Mg, 0.3 GTP-Na, 5 QX-314 bromide, and 10 HEPES; pH 7.35 with KOH, 280–300 mOsM. For current-clamp recordings the internal solution consisted of (in mM): 70 K-gluconate, 70 KCl, 5 NaCl, 1 MgCl_2_, 0.02 EGTA, 10 HEPES, 2 Mg_2_ATP, and 0.5 Na_2_GTP; pH 7.35 with trizma base, 280–300 mOsM. Recordings were done at a holding potential of −70 mV. Access resistance was monitored throughout the experiment. If access resistance varied >15% the experiment was discarded. For cell-attached recordings, gigaohm seals were made with (2–3 MΩ) micropipettes containing 150 mM NaCl and 10 mM HEPES. A concentric stimulating electrode (10 μm tip diameter) was placed ≈500 μm from the recorded cell. Electrical pulses (0.2 ms duration) were delivered by the stimulating electrode at a frequency of 0.02 Hz. The stimulus intensity was adjusted to evoke ≈100% of success in generating action currents in the recorded cell. For perforated patch recordings, micropipettes (2–3 MΩ) were filled with the same K-gluconate based solution supplemented with gramicidin (10 μg/ml). Once the gigaohm seal was established, approximately 15 min were necessary to get access to the recorded cell.

### Immunocytochemistry

Coronal slices containing the prefrontal cortex were fixed overnight with 4% paraformaldehyde/PBS solution (pH 7.4). The slices were then infiltrated with 30% sucrose and cut on a vibratome into 40 μm sections. Sections were incubated for 40 min in PBS solution containing 0.2% Triton X-100, then they were rinsed in PBS and incubated for 18–24 h at 4°C with primary rabbit anti-GABA_A_ receptor α4-subunit, α5-subunit, or δ-subunit antisera (diluted1:100) (Phosphosolutions, Aurora CO). After rinsing in PBS, sections were re-incubated for 2–4 h with secondary antibodies conjugated to fluorescein (diluted 1:100) (Vector Laboratories, Burlingame, CA). The reacted sections were first examined with an appropriate set of filters on an epifluorescence-equipped microscope. Afterwards, sections were mounted in an anti-quenching medium (Vectashield, Vector Laboratories) and examined under a confocal microscope (MRC 1024, Bio-Rad, Natford, UK) equipped with a krypton/argon laser. A two-line laser emitting at 488 nm wavelength was used for exciting fluorescein. Digitized images were transferred to a personal computer by using the image-capturing software (Confocal Assistant, T. C. Brelje, Minneapolis, MN). Omission of primary antisera resulted in no detectable signal (data not shown).

### Densitometry

Optical density was measured from color digitized images of the cells lebeled with anti-δ subunit antibody by using the program (Image-Pro Plus 6.1, Media Cybernetics, Silver Spring, MD). High power magnification images of labeled cells were used and optic density was determined by tracing density scanning lines through the soma of the cells (Morigaki and Goto, 2015).

### Western blot

Dissected tissue containing layer 5–6 from PFC were homogenized by ultrasonic treatment in a lysis buffer containing (in mM) 0.1 EDTA, and 10 Tris·HCl; pH 7.6, supplemented with an inhibitor cocktail (protease inhibitor cocktail, Roche). Protein concentration in samples was measured using the Lowry method (Lowry et al., 1951). For Western blot assays, 80 μg of protein were subjected to SDS-polyacrylamide electrophoresis, in a 7.5% SDS-PAGE and electro-blotted to PVDF membrane (Trans-Blot SD semi-dry electrophoretic transfer cell, Bio-Rad) and incubated with primary antibodies against different anti-GABA_A_ receptor subunits (1:1,000 dilutions). Membranes were then incubated with 1:10,000 peroxidase-conjugated secondary antibodies and antibody-antigen complexes were visualized using Luminate Crescendo Western HRP substrate (Millipore). Protein bands were visualized with a chemoluminescent reaction system (ChemiDoc, BioRad) and analyzed with the program Image lab (BioRad). The following antibodies were used in this study: goat anti-GABA_A_ receptor α4-subunit, α5-subunit, and δ-subunit antisera (Phosphosolutions, Aurora CO) and a secondary rabbit anti-goat HRP produced in rabbit (Sigma). Omission of primary antisera resulted in no detectable signal (data not shown).

### Drug incubation

In some experiments, slices were incubated in external solution bubbled with 95% O_2_-5% CO_2_ containing insulin (20 nM) for 1–2 h. In experiments using LY-294002, LY-303511, wortmannin, or genestein, the slices were incubated with one of these drugs for 1 h. Afterwards, insulin (20 nM) was added and 1 h more passed by before recordings. When protein synthesis inhibitors were used, the slices were incubated in cycloheximide or anisomycin for 40 min. Afterwards, insulin (500 nM) was added and the incubation lasted 30 more min before the recordings.

### Drug superfusion

In other experiments, SR-95531 (gabazine), 6-cyano-7-nitroquinoxaline-2,3-dione (CNQX), tetrodotoxin (TTX), gaboxadol (THIP), and L-655708 were directly applied into the bath solution. In some experiments, insulin was used at high concentrations (500 nM) to shorten the latency time of the effects. This made possible to evaluate the effects before and after insulin in the same cell (Wan et al., 1997; Vetiska et al., 2007). The drugs were dissolved into the bath saline from daily-made stock solutions and administered using a gravity-driven perfusion system. The time required for obtaining equilibrated concentrations of the drugs in the recording chamber was 3–4 min. For voltage-clamp recordings, at least 10 min were allowed for stabilization after breaking the membrane during which the blocker of glutamate receptor (CNQX) was present in the superfusion solution. Afterwards, SR-95531 (gabazine) was added and its effects were recorded during the administration (8–15 min). For cell attach experiments, insulin (500 nM) was bath applied during the cell recordings. In other set of experiments the slices were previously incubated in LY-294002 or LY-303511 for 1 h before insulin application.

Insulin, TTX, CNQX, gabazine, cycloheximide, anisomycin, gaboxadol (THIP), genistein, wortmannin, LY-294002 and LY-303511 were purchased from Sigma-Aldrich RBI (St. Louis, MO). L-655708 and gramicidin were purchased from Tocris Bioscience (Ellisville, MO).

### Statistical analysis

GABA_A_ receptor-mediated tonic current was measured as the resulting shift of the holding current when the GABA_A_ receptor antagonist gabazine (20 μM) was applied. For quantification, 5 ms long samples of the holding current were taken every 100 ms before and at the maximum effect of gabazine. The subtraction of the minor from the maximal current value was taken as the tonic current (Drasbek and Jensen, 2006). The resulting values were then normalized and used for statistical analysis. Offline analysis of the data was performed using Clampfit 10.2 (Molecular Devices) and graphing and statistical software (Origin 8, Microcal, Northampton MA). Data are expressed as means ± S.E.M. For each experimental group a minimum of 5 cells were recorded, 1 cell per slice. Statistical analysis was performed with GraphPad Prism 6 software (San Jose CA, USA) using Wilcoxon's or Mann-Whitney's tests for paired or non-paired samples respectively (*p* < 0.05 was taken as significant).

## Results

### Insulin modulates GABA_A_ activated currents in rat prefrontal cortex

**Figure 1:**
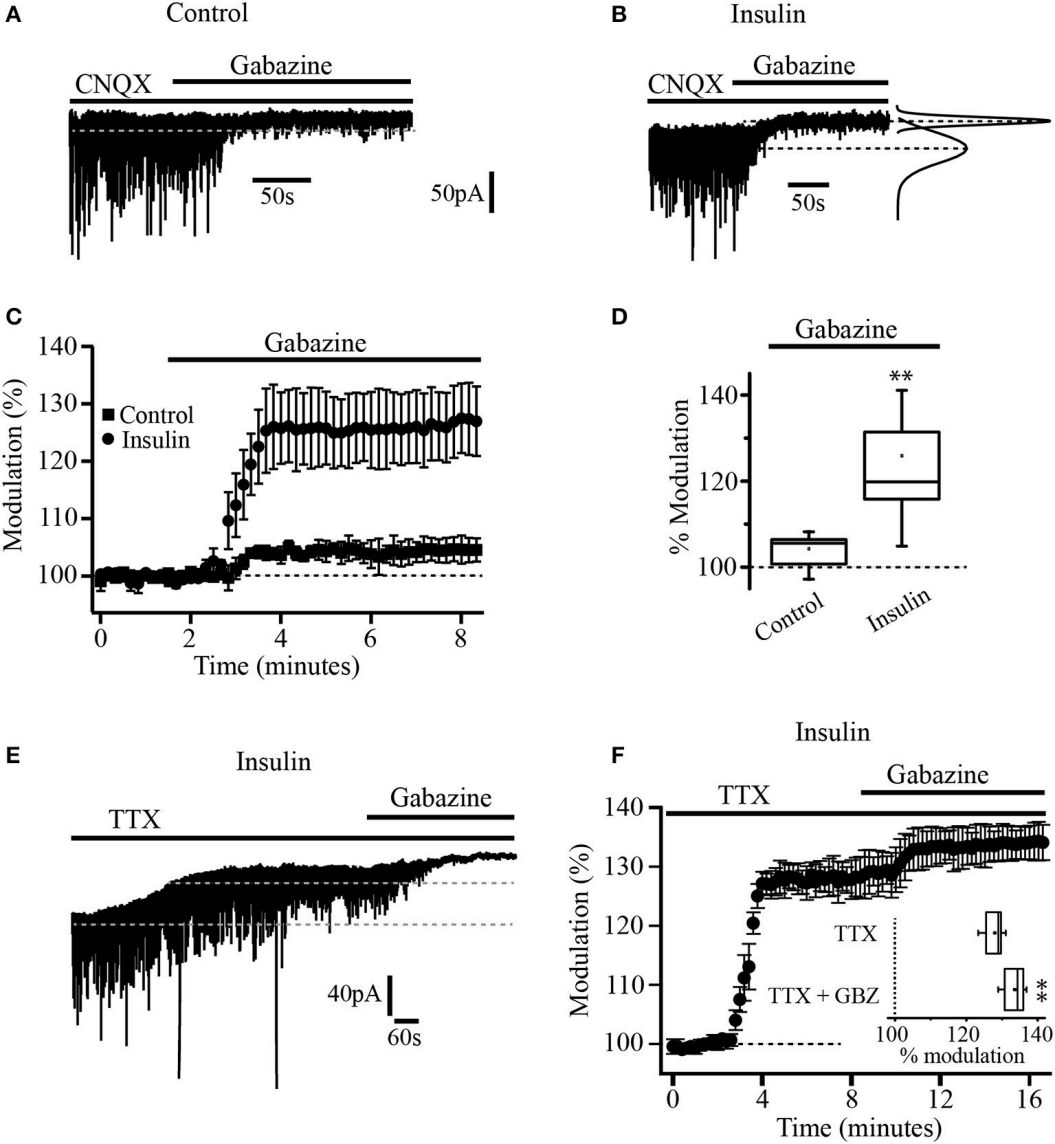
Insulin increases tonic current in PFC pyramidal neurons. **(A)** Current recording of a single cell in control conditions (without insulin). Black bars over the recording indicates the time application of CNQX (50 μM) and gabazine (SR-95531, 20 μM). Gabazine inhibits IPSCs and causes a small but significant shift of the holding current in control conditions (no insulin). (**B)** Current recording of a single cell from a slice previously incubated with insulin (20 nM). Gabazine blocked the IPSCs and produced a bigger shift of the holding current. At right, Gaussian fits of the histograms of 30 s current segments taken from the control period and after gabazine application are shown. The Gaussian peaks are indicated by dash lines. The lower dash line corresponds to the baseline current. The upper dashed line indicates the current level after gabazine application. **(C)** Graph that compares the response to gabazine of cells from control slices (dark squares) with cells from insulin-incubated slices (dark circles). The response is expressed as percentage of the basal current. **(D)** Box plot summary comparing the gabazine modulation in control (*n* = 5) and insulin conditions (*n* = 7). **(E)** Current recording of a single cell showing the effect of TTX (500 nM). Black bars over the recording indicates the time application of TTX and gabazine. **(F)** Temporal course of the effect of TTX and TTX plus gabazine. All TTX experiments were performed in cells from slices previously incubated with insulin and in the presence of CNQX. Inset in **(F)** shows the box plot summary comparing the effect of TTX and TTX plus gabazine (GBZ) (*n* = 5) ^**^p < 0.01.

A total of 116 neurons were recorded and assigned to the different experimental groups. First, we wanted to know whether insulin was able to modulate tonic currents in the PFC. We performed whole-cell patch-clamp recordings from layer 5/6 pyramidal neurons identified on the base of their morphology and regular firing pattern. The cells recorded had a resting membrane potential of 65 ± 2 mV and an input resistance (R_N_) of 186 ± 37 MΩ (*n* = 37). We used a Cs-based high chloride internal solution and all the experiments were done in the presence of CNQX (50 μM) to block AMPA/kainate glutamate receptors. The statistical tests were performed using the normalized data for all the experiments. The administration of the GABA_A_ selective antagonist, SR95531 (gabazine, 20 μM) in the bath solution suppressed the fast, inhibitory postsynaptic currents (IPSCs) and produced a small but statistically significant shift of the holding current by 4.9 ± 1 pA which represents 13.2 ± 1% with respect to the baseline (Figures 1A,C, black squares) (Wilcoxon test, *p* = 0.0022, *n* = 5). When the slices were incubated with insulin (20 nM) for 2 h, the application of gabazine produced a clear increase in the shift of the holding current by 38 ± 2.2 pA (25.6 ± 1.8%, *n* = 7) with respect to the baseline (Figures 1B,C, black circles). The box plot (Figure 1D) compares the holding current shift produced by gabazine in control conditions (non-incubated cells) vs. cells incubated with insulin (Mann-Whitney test, *p* = 0.0025). Similar results were observed by using bicuculline or picrotoxin (10 and 40 μM respectively, not shown). These first experiments suggested the presence of GABA_A_-mediated tonic current and its modulation by insulin in PFC pyramidal neurons. Since it is well-known that tonic currents depend on the ambient GABA that activates GABA_A_ extrasynaptic receptors (Bright et al., 2007), we wanted to know if the action potential-dependent vesicular release contributed to the ambient GABA in the PFC. To address this issue, pyramidal neurons of PFC were recorded in the presence of TTX (tetrodotoxin 500 nM) to inhibit action potential-dependent synaptic activity. In slices incubated with insulin, TTX produced a shift of the holding current by 39 ± 1.2 pA (27.9 ± 2.6%) with respect to the baseline. Under these conditions, the application of gabazine (20 μM) still was able to produce a small but statistically significant shift of the holding current (Figures 1E,F). The shift of the holding current produced by TTX plus gabazine was smaller (5.6 ± 1.4%) as compared with TTX alone. The box plot (Figure 1F, inset) compares the shift of the holding current produced by TTX alone vs. TTX plus gabazine (Wilcoxon test, *p* = 0.0065, *n* = 5).

### Insulin activates PI3K pathway

**Figure 2:**
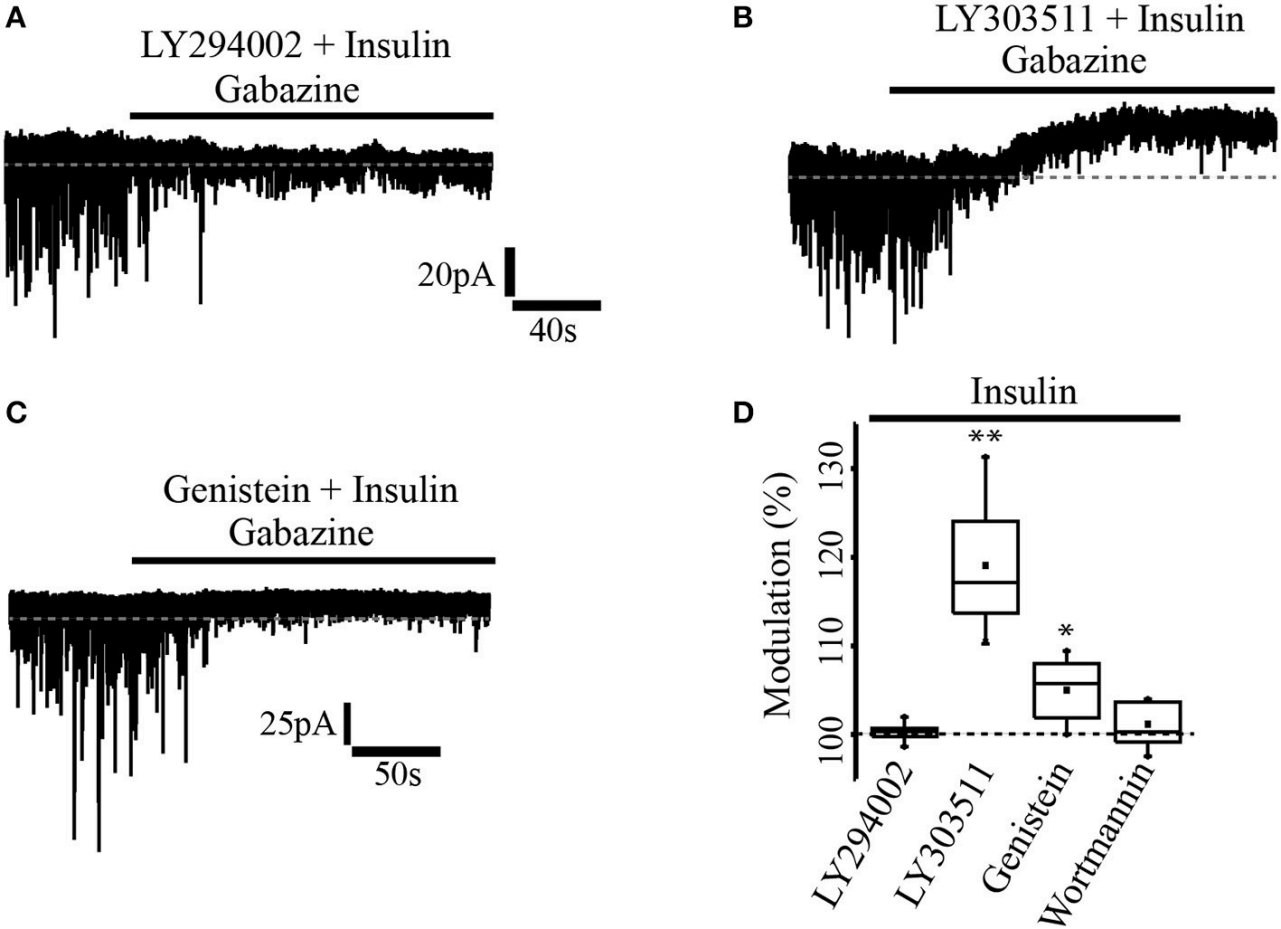
Insulin effect was mediated through PI3K/Akt pathway. **(A)** In slices previously incubated with the PI3K inhibitor, LY294002 (1 μM) and insulin, gabazine had no effect on the tonic current. **(B)** The incubation with insulin and the inactive analog of the PI3K inhibitor, LY303511 (5 μM) failed to prevent the shift of the tonic current induced by gabazine. **(C)** In slices incubated with insulin and genistein (50 μM), a tyrosine kinase inhibitor, gabazine produced a small effect on the tonic current. **(D)** Box plot summary showing the normalized shift produced by gabazine for LY294002 (*n* = 5), LY303511 (*n* = 7), genestein (*n* = 6), and wortmannin (200 nM, *n* = 5) ^*^p < 0.05, ^**^p < 0.01.

In a previous work (Vetiska et al., 2007) it was reported that insulin modulates GABA_A_ receptor expression by promoting the activation of the enzyme phosphatidylinositol-3-kinase (PI3K). To explore this possibility in PFC pyramidal neurons, one group of slices were incubated with insulin (20 nM) in the presence of the PI3K selective inhibitor, LY294002 (1 μM) before cell recordings (see methods). It was found that insulin modulation of GABA_A_ tonic current was completely suppressed by the PI3K inhibitor, that is, no effect on the holding current was observed after gabazine application (*n* = 5) (Figure 2A). Another group of slices was incubated with insulin and the inactive analog of the PI3K inhibitor, LY303511 (5 μM). When gabazine was applied, neurons recorded from slices incubated with insulin plus LY303511 exhibited a shift in the holding current by 27.4 ± 1.3 pA representing 19.6 ± 6% with respect to the baseline (*n* = 7) (Figure 2B). This effect was statistically significant when compared with the effect of gabazine on the LY294002 group (Mann-Whitney test, *p* = 0.0013). Wortmannin (200 nM), another PI3K inhibitor, also blocked the effect of gabazine on tonic current (*n* = 5, Figure 2D). On the other hand, when the cells were incubated with genistein, a broad-spectrum tyrosine kinase inhibitor, gabazine produced a small but statistically significant shift of the holding current by 9.3 ± 4.5 pA (5.01 ± 3.5%) with respect to the baseline (*n* = 6) (Wilcoxon test, *p* = 0.0331, Figure 2C).

### Tonic current is mediated by α5 and δ subunit-containing GABA_A_ receptors

**Figure 3:**
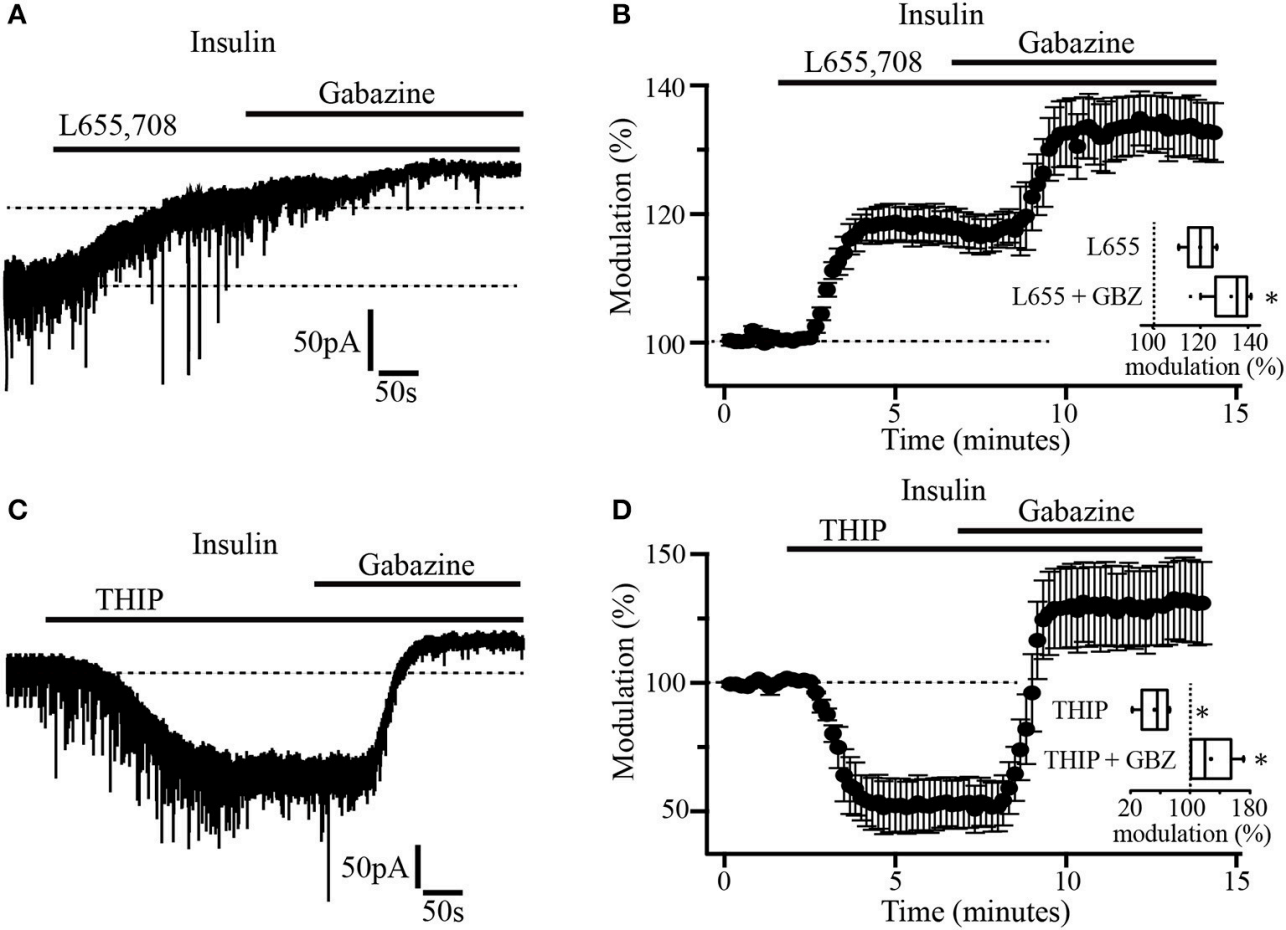
Insulin-sensitive tonic current is mediated by α5 and δ subunit-containing GABA_A_ receptors. **(A)** The α5 inverse agonist, L-655, 708 antagonizes GABA_A_ receptor-mediated tonic current in pyramidal neurons. Note that gabazine application caused an additional shift of the tonic current (upper dash line). **(B)** Shift of the tonic current by L-655, 708 and the subsequent application of gabazine expressed as percentage of the basal level (*n* = 7). The inset shows a box plot comparing L-655, 708 with L-655, 708 plus gabazine (GBZ). **(C)** THIP (5 μM) an agonist for δ subunit-containing GABA_A_ receptors, induces a downward shift in the holding current which is then blocked by gabazine application. **(D)** Shift of the tonic current by THIP and gabazine application expressed as percentage of the basal level (*n* = 5). The inset shows a box plot summary of the results. These experiments were performed in slices incubated with insulin (20 nM) ^*^p < 0.05.

As previously reported, α5 and δ subunits are more commonly expressed in GABA_A_ extrasynaptic receptors (Scimemi et al., 2005). To explore which type of subunits constitute the GABA_A_ receptors mediating tonic currents in PFC pyramidal neurons, we first examined the effect of L655, 708, an inverse agonist with high selectivity for GABA_A_ receptor containing the α5 subunit. The drug was applied to slices previously incubated with insulin (20 nM). In these conditions, the bath application of L-655, 708 (5 μM) by itself, induced an upward shift of the holding current by 41.19 ± 1.8 pA which represents 18.7 ± 0.6% with respect to the baseline. The subsequent administration of gabazine produced an additional shift of the holding current by 17.11 ± 2.5 pA (13.02 ± 1.6%) with respect to L-655, 708 alone (Figures 3A,B). This suggests that the tonic current in PFC does not only depend on α5-containing GABA_A_ extrasynaptic receptors. Inset box plot (Figure 3B) compares the holding current shift produced by L655, 708 alone vs. L655, 708 plus gabazine (Wilcoxon test, *p* = 0.0156, *n* = 7). Afterwards, we tested the effect of THIP (gaboxadol), an agonist of GABA_A_ receptors containing the δ subunit. At a concentration of 5 μM, THIP produced a robust increase of the tonic current, revealed by a downward shift of the holding current by 171.33 ± 7.9 pA (Figure 3C). The addition of gabazine caused a big upward shift by 94.0 ± 9.8 pA that overpassed the initial holding current (black dotted line, Figure 3C). The normalized shift of the holding current with respect to the baseline, was (46 ± 2.1%, downward, *p* = 0.0313) and (29.2 ± 3.3%, upward, *p* = 0.0323) for THIP and THIP plus gabazine, respectively (Wilcoxon test, *n* = 5) (Figure 3D and inset boxplot).

### Insulin increases the expression of GABA_A_ extrasynaptic receptors

**Figure 4:**
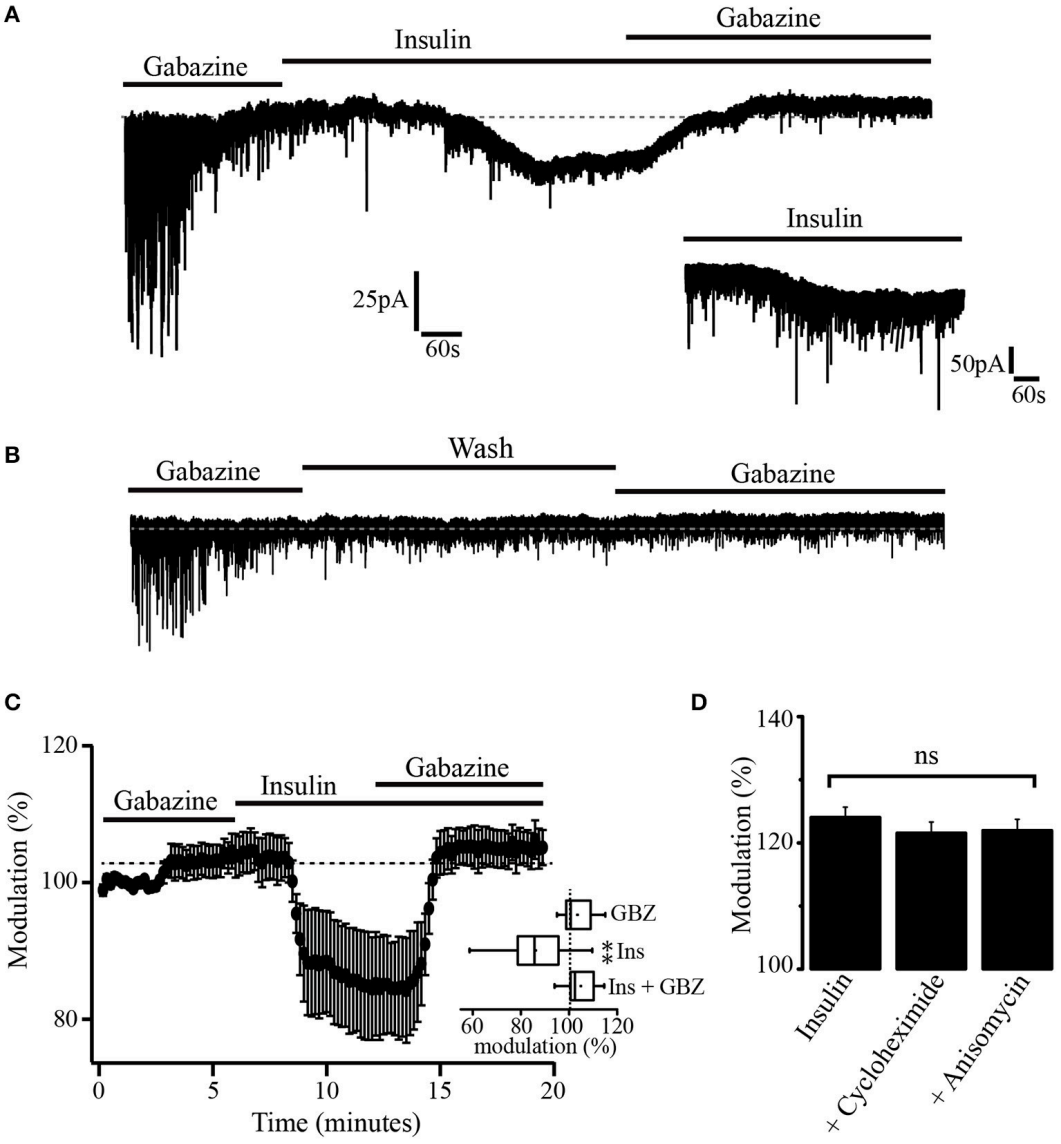
Insulin promotes GABA_A_ mediated tonic current. **(A)** After the blockage of GABA_A_ receptors with gabazine, insulin (500 nM) produced a downward shift of the holding current. This effect was reversed by a new application of gabazine. The inset is a 10 min current trace showing the effect of insulin alone. **(B)** When insulin was replaced by the vehicle (wash) a new administration of gabazine was without effect (*n* = 5). **(C)** Graph showing the shift of the tonic current produced by insulin normalized with respect to the effect of gabazine alone (*n* = 7). The inset shows a box plot graph comparing insulin and insulin plus gabazine (GBZ) vs. gabazine alone. **(D)** Bar plots comparing the normalized shift of the holding current by gabazine in cells from slices incubated with insulin alone (*n* = 5), insulin plus cycloheximide (*n* = 5), and insulin plus anisomycin (*n* = 5). Changes were not statistically significant (ns) ^**^p < 0.01.

Previous experiments have demonstrated that insulin promotes the translocation of GABA_A_ receptors from the cytoplasm to the synaptic membrane of hippocampal cells (Wan et al., 1997). To explore whether insulin could also increase the expression of extrasynaptic GABA_A_ receptors in membrane sites, we took advantage of the fact that, when gabazine is administered at a concentration of 20 μM for 10 min, its effects on the GABA_A_-mediated tonic currents do not revert 1 h after its application. Thus, we used gabazine (20 μM) to produce a long-term blocking of GABA_A_ receptors. Gabazine was applied for 15 min. Then, the GABA_A_ blocker was withdrawn and insulin (500 nM) was added to the bath solution. This high concentration of insulin was used to shorten the latency time of its effects (Wan et al., 1997; Vetiska et al., 2007). Insulin produced a downward shift of the holding current (46.36 ± 1.9 pA). Still in the presence of insulin, a second application of gabazine caused an upward shift of the holding current even when GABA_A_ receptors had been previously blocked (Figure 4A). The inset shows a 10 min trace of the effect of insulin alone (500 nM) in another neuron. As expected, the effect of insulin does not revert after 40 min of recording (not shown). Note that noise is increased after bath application of insulin alone which suggests that activation of extrasynaptic GABA_A_ receptors contribute to the basal noise. No effect was observed when insulin was replaced by the vehicle and an additional application of gabazine was made in 5 of 5 cells (Figure 4B). The normalized shift of the holding current produced by insulin with respect to gabazine alone was 18.3 ± 4.4% (*p* = 0.0011, Wilcoxon test, *n* = 7). The effect of insulin plus gabazine was not statistically different from the effect of gabazine alone (*p* = 0.0647, Wilcoxon test) (Figure 4C, inset). To test whether the effect of gabazine on the tonic current was due to new synthetized GABA_A_ receptors, we performed experiments in which the PFC slices were incubated separately with insulin alone (500 nM) or insulin plus the protein synthesis inhibitors, cycloheximide (50 μM) or anisomycin (40 μM) (see methods). In all these conditions, gabazine still was able to produce a shift of the holding current (Figure 4D). The shift of the holding current was 43.7 ± 8 pA (24.52 ± 1.6%), 31.4 ± 2 pA (23.54 ± 1.6%), and 26.2 ± 4 pA (22.95 ± 1.4%) for control (insulin alone, *n* = 5), insulin plus cycloheximide (*n* = 5), and insulin plus anisomycin (*n* = 5) respectively. No statistical differences were found when protein inhibitors groups were compared with the control (*p* = 0.1847 and *p* = 0.0543, Wilcoxon test) for cycloheximide and anisomycin, respectively. These data suggest that the insulin-induced tonic current does not depend on the synthesis of newly expressed GABA_A_ extrasynaptic receptors.

### Insulin decreases neuronal excitability

**Figure 5:**
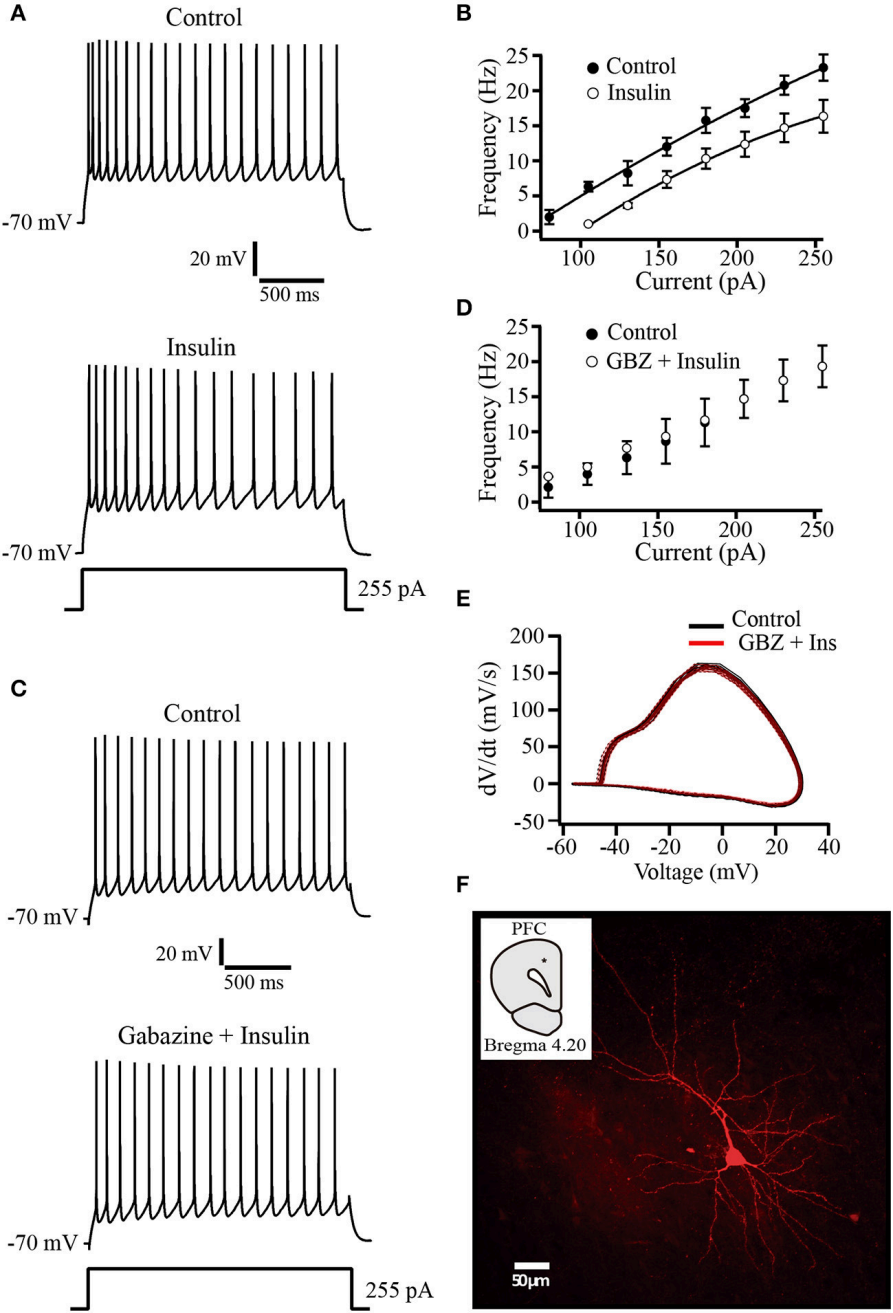
Insulin decreases the firing frequency evoked by depolarizing current injection. **(A)** Current clamp recordings in control (top) and insulin (500 nM, bottom) showing the response to a depolarizing current injection of 255 pA. Note that following insulin incubation, the frequency of action potential is decreased (bottom trace). **(B)** Current-frequency relationship in control and insulin conditions (top) (*n* = 5). **(C)** Current clamp recordings in control (top) and insulin plus gabazine (GBZ, bottom). Insulin has no effect on the firing frequency in the presence of gabazine (*n* = 6) (bottom trace). **(D)** Current-frequency relationship of control (filled circles and insulin plus gabazine (empty circles, top). **(E)** Phase-plot analysis of the action potential for both conditions. Control (black line); gabazine (SR) plus insulin (red line). **(F)** Typical PFC pyramidal neuron labeled with biocytin. The inset shows the recording site (black dot).

The observation that tonic GABA_A_ currents are increased by insulin raises the question about the physiological role of this modulation. To evaluate whether this insulin effect has an impact on neuronal excitability, we performed current clamp experiments by using a K-gluconate internal solution. Recorded pyramidal cells were subjected to increasing steps of depolarizing current injections from a holding potential of −70 mV. Afterwards, insulin (500 nM) was perfused during 10–15 min before repeating the current injection protocol. In all the cells tested, insulin produced a hyperpolarizing shift of ≈4 mV of the holding potential and a decrease of the firing frequency. Even when depolarizing current was injected to return the holding potential to −70 mV, insulin produced a reduction of the firing rate (Figures 5A,B). The normalized decrease of the firing frequency by insulin with respect to the control was 28.18 ± 9.5% at the maximal depolarizing pulse (255 pA) (Wilcoxon test, *p* = 0.008, *n* = 5). As comparison, in other experiments insulin was applied in the presence of gabazine (20 μM). Under these conditions, insulin had no effect on the firing frequency or the holding potential (*n* = 6) (Figures 5C,D). To determine whether insulin changed voltage-dependent currents we constructed phase plots of d*V*m/d*t* against *V*m from action potentials obtained before and after insulin in the presence of gabazine. The Figure 5E shows that phase plots obtained in both conditions overlapped each other, suggesting that insulin does not alter action potential involved conductances. The Figure 5F illustrates a typical pyramidal neuron from layer 5–6 PFC labeled with biocytin.

### Insulin decreases the gain of pyramidal PFC neurons

**Figure 6:**
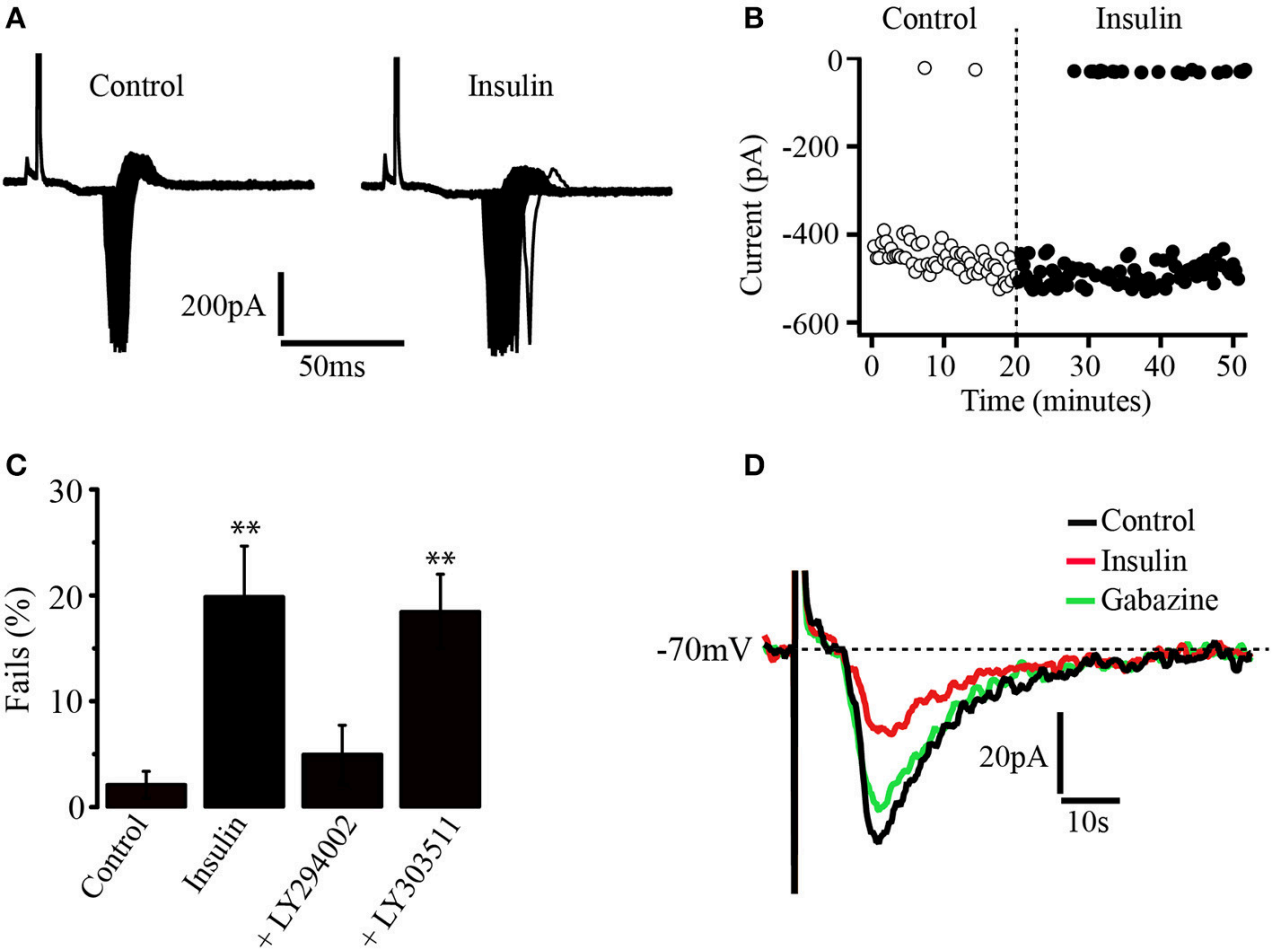
Insulin decreases the gain of pyramidal PFC neurons. **(A)** Recordings of action currents in cell-attached configuration in the absence (left) or the presence (right) of insulin (500 nM). Note the variability of the latency induced by insulin. **(B)** Insulin increases the number of fails in generating action currents (black dots) as compared with control (white dots). **(C)** Bar graphs show the fails percentages in control (*n* = 6), insulin (*n* = 6), the PI3K inhibitor LY294002 (*n* = 5), and the inactive analog LY303511 (*n* = 6). Only insulin and insulin plus LY303511 were statistically different from control. **(D)** Insulin (red line) decreases the amplitude of evoked synaptic currents and the effect is reversed by gabazine (green line) in perforated patch configuration (*n* = 5) ^**^p < 0.01.

The influence of insulin on neuronal excitability was tested in a more physiological approach. Thus, concentric electrodes (10 μm diameter) were placed within the PFC about 500 μm above from the recording site. Square wave pulses were delivered and evoked fast action currents underlying action potentials were recorded in loose cell-attached configuration from pyramidal neurons (see methods). The stimuli intensity was adjusted to have a success rate close to 100% of the response. It was observed that insulin (500 nM) increased the variability of the latency to the generation of the evoked currents which resulted into a greater spread of the response than in control conditions (Figure 6A). In addition, the rate of fails for evoked currents increased (Figure 6B). These effects of insulin were suppressed by previous incubation in the PI3K inhibitor, LY294002 but not by the inactive analog LY303511 (see methods). In the Figure 6C, the bar graph compares the fails of the different experimental groups with the control (no insulin group, *n* = 6). The percentages of fails were 19.8 ± 4.7%, 4.9 ± 2.7%; 18.4 ± 3.5% for insulin (*n* = 6), insulin plus LY294002 (*n* = 5), and insulin plus LY303511 (*n* = 6), respectively (Figure 6C). Only insulin and insulin plus LY303511 groups were significantly different from the control group (*p* = 0.0320 and *p* = 0.0313, Wilcoxon test) respectively. In another set of experiments a subthreshold stimulus was applied within the PFC and the evoked excitatory synaptic response was recorded in perforated patch mode from pyramidal neurons (see methods). Bath application of insulin (500 nM) decreased the amplitude of the evoked synaptic response and the effect was reversed by gabazine. The decrease of the evoked synaptic current, as compared to the control was 57.8 ± 2%, (Wilcoxon test, *p* = 0.0062, *n* = 5) (Figure 6D).

### Insulin promotes an increase of extrasymaptic GABA_A_ receptors in the cell membrane

**Figure 7:**
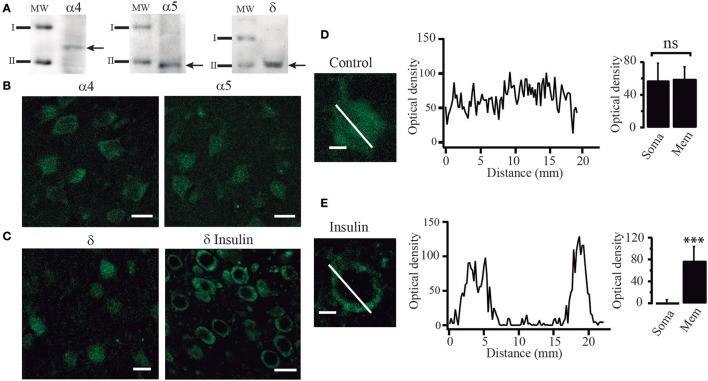
Insulin promotes the translocation of GABA_A_ receptors from the cytoplasm to the cell membrane. **(A)** Western blot analysis showing immunoreactive products of tissue samples from layer 5–6 of PFC. Arrows indicate bands recognized by the anti-α4 antibody (left), anti-α5 antibody (middle), and anti-δ antibody (right), corresponding to protein with masses of 61, 49, and 50 KDa, respectively (I = 75 KDa, II = 50 KDa). **(B)** Immunoreactivity to α4 (left) and α5 (right) GABA_A_ receptor subunits. **(C)** Immunoreactivity to δ receptor subunit in cells from control slices (left) and cells from slices incubated with insulin (δ insulin, right). Note that in the cells incubated with insulin, staining was stronger in the boundaries as compared with the control cells **(D)** High magnification image of a single non-incubated cell (control) stained with an anti-δ antibody (left). The graph in the middle shows the optical density measured through the length of the white line (distance). Optical density had small variations along the cell. Bar graphs indicate no difference of the optical density of the membrane (Mem) with respect to the soma in 24 cells (right). **(E)** A single insulin-incubated cell stained with an anti-δ antibody (left). The graph in the middle shows the optical density measured through the length of the cell (white line). The graph shows two peaks of optical density representing the white line crossing the limits. Note that when the white line crosses the soma the optic density is almost zero. Bar graphs indicate the difference in the staining of the membrane (Mem) with respect to the soma in 28 cells (right). Scale in **(B,C)**, 20 μm. Scale in **(D,E)**, 5 μm. ^***^p < 0.001.

Since our experiments suggested that tonic currents were mediated by α5 and δ containing GABA_A_ receptors, we searched for the presence of these proteins by using Western blot analysis and immunocytochemical techniques. Western blot analysis identified α4, α5, and δ immunoreactive products in protein samples obtained from layers 5–6 of PFC tissue. We detected bands corresponding to proteins with masses of 61, 49, and 50 kDa as expected for α4, α5, and δ subunits respectively (Khrestchatisky et al., 1989; Shivers et al., 1989; Malherbe et al., 1990) (Figure 7A). In agreement with these data, immunocytochemistry experiments showed that PFC cells were labeled with α4, α5, and δ polyclonal antibodies (Figures 7B,C). In some experiments, the δ subunit antibody was probed in slices maintained in control conditions and compared to slices incubated with insulin (100 nM) (Figures 7C–E). The cells from control slices exhibited a diffuse anti-δ labeling through the soma of the cell as shown at higher power magnification (Figure 7D, left). In contrast, the cells from insulin-treated slices exhibited a strong labeling of the cell limits and weak staining of the soma (Figure 7E, left). This data supports the idea that insulin promotes the trafficking of GABA_A_ receptors to the membrane. Optical density did not change much along the cell axis in cells from non-incubated slices (Figure 7D, middle and right graphs, *p* = 0.4524, Wilcoxon test, *n* = 24). On the other hand, cells from insulin incubated slices showed higher optical density in the limits as compared to the soma (Figure 7E). Two peaks, corresponding to the optical density measured in the limits of the cell are apparent in the Figure 7E (middle graph) while the optical density in the soma is close to zero (*p* = 0.0001, Wilcoxon test, *n* = 28). Similar data were obtained by using a polyclonal anti-α4 antibody (not shown).

## Discussion

One of the main findings of the present study was that insulin increases GABA_A_-mediated tonic currents in layers 5–6 of PFC. This is the first time that insulin effects on tonic currents are tested in the PFC, a structure involved in cognitive, mood, and motor behaviors. Our data add to others reporting the presence of GABAergic tonic currents in different cortical regions of both, humans and rodents (Yamada et al., 2004; Drasbek and Jensen, 2006; Scimemi et al., 2006; Sebe et al., 2010).

Insulin promotes the trafficking of extrasynaptic GABA_A_ receptors to the cell membrane. This insulin-mediated trafficking mechanism was previously described for synaptic GABA_A_ receptors in HEK cells and hippocampal cultured neurons (Wan et al., 1997; Wang et al., 2003; Vetiska et al., 2007). In addition, we determined the presence of α4, α5, and δ proteins in layers 5–6 of PFC. These proteins are known to form extrasynaptic GABA_A_ receptors which produce tonic inhibitory currents in different brain areas.

This work also showed that GABA_A_-mediated tonic currents are exerting a constant inhibition on pyramidal PFC neurons which became evident when the sole application of gabazine, a GABA_A_ receptor antagonist, produced a small but significant shift of the tonic current (see Figure 1).

In the past, it was reported that gabazine failed to block GABA_A_-mediated tonic currents (Bai et al., 2001; Semyanov et al., 2003). However, in our experiments and in other, more recent studies (Drasbek and Jensen, 2006; Drasbek et al., 2007; Vardya et al., 2008; Jin et al., 2011) a consistent blockage of the GABA_A_-mediated tonic currents by gabazine was observed. These differences may be explained by the level of expression of extrasynaptic GABA_A_ receptors. Also, different subunit combinations forming GABA_A_ extrasynaptic receptors could result in different pharmacological properties of gabazine.

### Two separate types of extrasynaptic GABA_A_ receptors mediate tonic currents in PFC

Our data indicate that extrasynaptic GABA_A_ receptors of PFC are constituted by α5 and δ subunits because L-655, 708, an inverse agonist with high selectivity for GABA_A_ receptors containing the α5 subunit, blocked the tonic currents while gaboxadol (THIP), whose effects generally correlate with the expression of δ subunit increased these currents. Accordingly, immunocytochemical experiments and Western blot analysis confirmed the presence of α5 and δ GABA_A_ subunits in layers 5–6 of PFC. Most likely, α5 and δ subunits are forming different types of GABA_A_ receptors, since we found that L-655, 708 only partially blocked the insulin-induced tonic current. The α5 subunit usually forms α5β1-3γ2 GABA_A_ extrasynaptic receptors as in hippocampal pyramidal cells (Brünig et al., 2002; Farrant and Nusser, 2005). On the other hand, the δ subunit forms receptors with the α4 in several forebrain regions including the neocortex (Barnard et al., 1998; Farrant and Nusser, 2005). Furthermore, co-immunoprecipitation studies have shown that antibodies against the δ subunit precipitate the α4 subunit in thalamic tissue (Jia et al., 2005). In this work, Western blot analysis showed that apart from α5 and δ, α4 protein was also present in PFC tissue. Then, it is possible that combined α4 and δ subunits would form a separate type of extrasynaptic GABA_A_ receptor in layers 5–6 of PFC.

### Most of ambient GABA depends on action potential-mediated release in PFC

Several sources are thought to contribute to the ambient GABA that generates inhibitory tonic currents. These include action potential-mediated release (Brickley et al., 1996; Bright et al., 2007; Glykys and Mody, 2007) as well as non-synaptic release such as reverse transport (Richerson and Wu, 2003) and astrocytic release (Volknandt, 2002; Rossi et al., 2003; Kozlov et al., 2006). Our experiments showed that action potential-dependent release is the main source of ambient GABA in layers 5–6 of PFC although other mechanisms also contribute. This idea is based on the fact that TTX reduced most of the tonic current and the subsequent application of gabazine produced an additional but smaller reduction of the current in the presence of insulin. TTX has also been shown to cause a great reduction of tonic conductance in cultured neurons from the hippocampus and cerebellum (Leao et al., 2000; Petrini et al., 2004). However, in mature cerebellar granule cells most of the tonic conductance is action potential independent (Wall and Usowicz, 1997; Rossi et al., 2003).

### Insulin effects are mediated through activation of tyrosine kinase receptors and PI3K/Akt pathway

The effect of insulin on the tonic current was mediated through the activation of PI3K/Akt signaling pathway since the PI3K blockers LY294002 and wortmannin suppressed the shift of the holding current induced by gabazine in insulin-incubated neurons. It was observed that the shift of the holding current was strongly reduced by genistein, a broad-spectrum tyrosine kinase inhibitor, indicating a specific effect of insulin through the activation of tyrosine kinase receptors. It is well-known that insulin or insulin grow factor bind to their receptors whose intrinsic tyrosine kinase activity phosphorylates the intramembrane domains that serve as docking site for insulin receptor substrate (IRS) leading to the activation of PI3K and Akt proteins (Wozniak et al., 1993; Bassil et al., 2014).

### Insulin decreases the excitability of layer 5–6 pyramidal PFC neurons

Three different experimental protocols were performed to assess the effect of insulin on cellular excitability. In current clamp recordings, insulin produced a negative shift of the holding potential and decreased the firing frequency evoked by current injection in all the cells tested. This effect was abolished when GABA_A_ receptors were previously blocked by adding gabazine to the bath solution. Insulin could change the firing frequency by modulating other conductances. For instance, it has been reported that insulin or insulin-like growth factor (IGF-1) inhibit Kv1.3 potassium channels (Bowlby et al., 1997; Fadool et al., 2000) and increase the expression of Ca^2+^ channels (Viard et al., 2004; Toledo et al., 2012). Moreover, PI3K signaling has been found to modulate persistent Na^+^ currents in cardiac myocytes (Lu et al., 2012). Insulin modulation of these conductances would modify the dynamic of the action potentials (APs). Thus, changes in persistent Na^+^ current would shift the AP threshold (Mercer et al., 2007). On the other hand, inhibition of the Kv1.3 channels would increase the excitability and oppose to AP repolarization. Also, an increase of Ca^2+^ influx would lead to the activation of Ca^2+^-dependent K^+^ channels changing the duration and amplitude of the AP afterhyperpolarization. The possibility that any of these conductances were involved in the effect of insulin on PFC pyramidal neurons was discarded because phase plots (dV_m_/dt against V_m_) obtained from APs before and after insulin, were identical.

To have a more physiological approach, we also performed cell attached experiments to avoid dialyzing the cell. In these conditions, insulin increased the fails of action currents evoked by local stimulation and caused a variation in the latency (see Figure 6). Lastly, patch perforated experiments showed that insulin decreased the amplitude of excitatory evoked synaptic potentials and gabazine reversed this effect. Together, these data indicate that insulin reduces the excitability of layer 5–6 PFC neurons and that an increase of GABA_A_-activated tonic currents is involved in this effect.

### Insulin promotes the expression of extrasynaptic GABA_A_ receptors to the membrane

After the blockage of GABA_A_ receptors with gabazine (20 μM), insulin still produced a downward shift of the current which was reverted by a new administration of gabazine (Figure 4). This suggests that insulin produced the expression of new GABA_A_ receptors, since in our experiments, the effect of gabazine did not revert 1 h after its application. A reversible effect of gabazine has previously been reported for synaptic GABA_A_-mediated currents (Bai et al., 2001). However, it might be that extrasynaptic GABA_A_ receptors have higher affinity for gabazine than the synaptic ones. Then, it would take more time to wash the effect of gabazine on the tonic currents.

**Figure 8:**
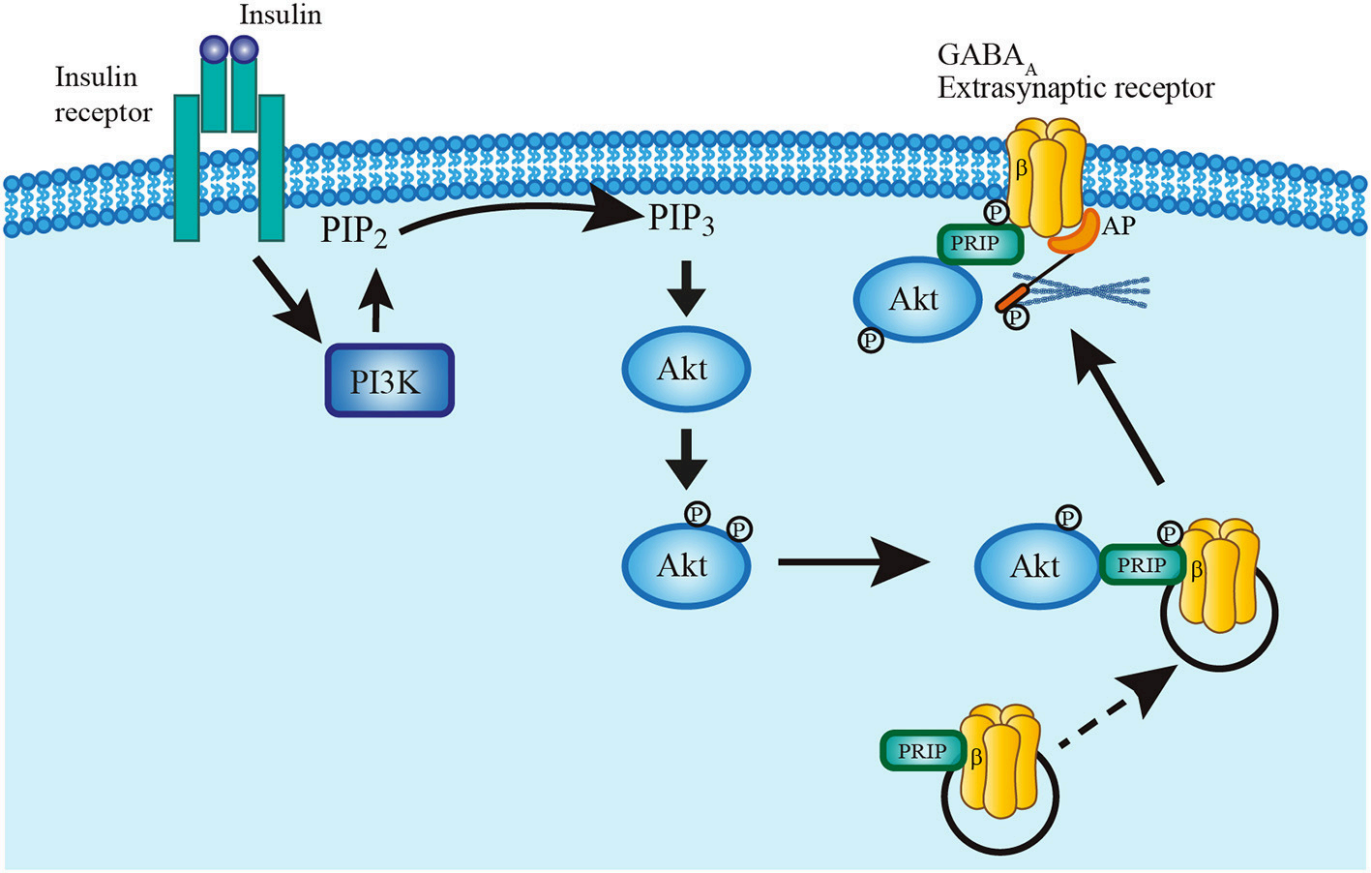
Model describing insulin mechanism of GABA_A_ receptor translocation. Insulin binding to its receptor activates PI3K pathway which in turn phosphorylates PIP2 converting it to PIP3. PIP3 recruits Akt into the plasma membrane to rapidly phosphorylate GABA_A_ receptors at the β subunit. Phospholipase-C-related catalytically inactive protein (PRIP) has a key role in the translocation mechanism by coupling Akt and phosphorylating GABA_A_ receptors. The complex is then translated and fixed to the plasma membrane by mean of an anchoring protein (AP) which also binds to the cytoskeleton (Hausrat et al., 2015).

It is unlikely that insulin acts by promoting the synthesis of new GABA_A_ receptors since the protein synthesis inhibitors cycloheximide and anisomycin did not prevent the insulin-dependent increase of GABA tonic currents. Rather, it is possible that insulin leads to the formation of the PI3K-GABA_A_ receptor complex which plays an essential role in the expression of GABA_A_ synaptic receptors in the membrane of hippocampal neurons (Wan et al., 1997; Vetiska et al., 2007). This hypothesis is reinforced by the immunocytochemical data that show an increase of anti-δ subunit staining in the limits of the cell as compared with the soma after insulin incubation (see Figure 7). The Figure 8 shows a scheme of the trafficking mechanism of the GABA_A_ extrasynaptic receptors. Once the receptors are phosphorylated via PI3K/Akt pathway, the complex is transferred to the cell membrane where it is recognized by an anchorage protein in the extrasynaptic domain (Vetiska et al., 2007; Hausrat et al., 2015).

### Physiological relevance

Insulin effects in the brain have important physiological implications because they increase tonic inhibition and alter membrane cell properties such as time constant and input resistance. These effects can modify the resting membrane potential, the firing rate and the neuronal firing pattern (Mitchell and Silver, 2003; Semyanov et al., 2003; Rothman et al., 2009). Impaired insulin signaling, for instance in diabetes mellitus, increases the risk of epilepsy (Verrotti et al., 2008; Ramakrishnan and Appleton, 2012) and cognitive disabilities (Seaquist, 2010; Kullmann et al., 2016). Until now, the link between insulin signaling and these pathologies is poorly understood but there are some clues. For instance, reduced or aberrant expression of extrasynaptic α5 and δ-containing GABA_A_ receptors is associated with epileptogenesis (Schwarzer et al., 1997; Houser and Esclapez, 2003; Dibbens et al., 2004,?; Peng et al., 2004). On the other hand, enhanced expression of δ-containing GABA_A_ receptors reduces anxiety and seizure susceptibility (Maguire et al., 2005; Maguire and Mody, 2007). In the hippocampus, CA1 pyramidal neurons have α5-cointaining GABA_A_ receptors at the dendrites. The tonic current through these channels can cause shunting inhibition and limit the effects of excitatory inputs to these neurons. Behavioral experiments in mice have shown that deletion or inhibition of α5 GABA_A_ receptors in hippocampus correlates with improved memory (Caraiscos et al., 2004; Martin et al., 2010). This can be explained because deletion of these channels increases the input resistance of CA1 neurons. This facilitates long-term potentiation (LTP) and improves memory performance. Then, an increase of the levels of insulin in the brain would have important physiological implications. The enhancing of tonic currents by insulin would decrease neuronal excitability. This could induce alterations, not only of memory, but of other cognitive processes as well. Insulin inhibition of PFC neuron excitability could produce alterations of functions such as memory, cognition and mood. Accordingly, a previous work reported that insulin stimulates cortical beta and theta activity and these effects are reduced in patients with insulin resistance (Tschritter et al., 2007). More extensive studies are necessary to explore the effects of insulin on GABA_A_-receptor mediated tonic currents and its relationship with several illnesses. Our data, as well as the data from other studies, suggest that extrasynaptic GABA_A_ receptors could be strategic targets for the treatment of some pathologies related to altered insulin signaling in the brain.

## Author contributions

ST-R performed the experiments, analyzed data, and wrote the paper. DC-R, DT, and SM analyzed data. EG designed the experiments. GA-L performed molecular experiments. SH-L designed the experiments and wrote the paper.

### Conflict of interest statement

The authors declare that the research was conducted in the absence of any commercial or financial relationships that could be construed as a potential conflict of interest.
